# Single-tube multiplex real-time PCR with EvaGreen and high-resolution melting analysis for diagnosis of α^0^-thalassemia--^SEA^,--^THAI^, and--^CR^ type deletions

**DOI:** 10.1371/journal.pone.0293838

**Published:** 2023-11-06

**Authors:** Chedtapak Ruengdit, Manoo Punyamung, Nutjeera Intasai, Sakorn Pornprasert

**Affiliations:** 1 Department of Medical Technology, Faculty of Associated Medical Sciences, Chiang Mai University, Chiang Mai, Thailand; 2 Associated Medical Sciences Clinical Service Center, Faculty of Associated Medical Sciences, Chiang Mai University, Chiang Mai, Thailand; Shoklo Malaria Research Unit, THAILAND

## Abstract

Regions with a high prevalence of α-thalassemia (α-thal) require simple, rapid, and accurate tests for carrier screening and prenatal diagnosis. Diagnosis of multiple deletions in a single tube is necessary to clearly identify individuals with α^0^-thalassemia in the routine setting, especially in at-risk couples. Therefore, we aimed to develop a single-tube multiplex real-time PCR with EvaGreen and high-resolution melting (HRM) analysis for the identification of α^0^-thalassemia Southeast Asian (SEA), Thai and Chiang Rai (CR) type deletions. The results of the HRM analysis indicated that the amplified fragments from α^0^-thal--^CR^,--^THAI^,--^SEA^, and the wild-type α-globin gene had specific peak heights at mean melting temperature (T_m_) values of 85.40°C, 86.50°C, 87.65°C, and 91.04°C, respectively. The frequencies of α^0^-thal--^SEA^,--^THAI^,--^CR^ obtained from routine testing in 2,135 samples were 17.89%, 0.19% and 0.19%, respectively. This method would be useful for preventing Hb Bart’s hydrops fetalis. Detection of multiple deletions in a single run is cost-effective, highly accurate and timesaving. This technique could enable wider α-thalassemia diagnosis in high prevalence areas and served as an example for thalassemia routine setting.

## Introduction

**Fig 1:**
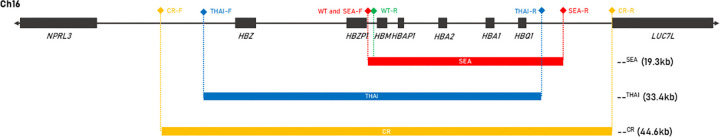
Schematic representation of α^0^-thal--^SEA^,--^THAI^ and--^CR^ type deletions on α-globin gene cluster and designated primers. Abbreviations: SEA, Southeast Asian; CR, Chiang Rai; F, Forward primer; R, Reverse primer; WT, Wild-type; kb, kilobases.

Alpha-thalassemia (α-thal) is a genetic hematological abnormality that is frequently found in tropical and subtropical regions. The deletion of one (-α) or both (—) α-globin genes on chromosome 16p13.3 [1, 2] has been reported in more than 95% documented cases of α-thalassemia [3]. These gene deletions cause mild α^+^-thal and severe α^0^-thal. The most common mutations of α^0^-thal in the Thai population are the Southeast Asian (--^SEA^; NC_000016.9:g.215396_234699) and Thai (--^THAI^, NC_000016.9:g.199862_233311) type deletions. The--^SEA^ deletion is approximately 19.3 kb in length, involving both functional α-globin genes but leaving the ζ2-globin gene intact [4, 5, Fig 1] and the--^THAI^ type deletion, is approximately 33.4 kb in length, involving both α-globin genes as well as the ζ2 gene [6, Fig 1]. In Southeast Asia, the carrier frequencies of α^0^-thal--^SEA^ and--^THAI^ type deletions were 4–14% and 0.2%, respectively, depending on the population [5, 7, 8].

Recently, a novel α^0^-thal 44.6 kb deletion (—^44.6^; NC_000016.9:g.194,214_238,840), first described in southern China [9, Fig 1], has been identified in northern Thailand, where it is known as the Chiang Rai deletion (--^CR^) and was found at a frequency of 1.71% in a specific population [10, 11]. Similar to other α^0^-thal carriers, the--^CR^ carriers displayed asymptomatic disease with apparent microcytic red blood cells. However, compound heterozygosity of this mutation and--^SEA^ or -α^3.7^ can cause Hb Bart’s hydrops fetalis and HbH disease, respectively [10, 12]. Although carriers of α^0^-thal do not show any clinical symptoms, couples in which both individuals are carriers have a 25% chance of conceiving a fetus that is homozygous for the deletion, which manifests as Bart’s hydrops fetalis, the most severe thalassemic syndrome. All these fetuses either die *in utero* or soon after birth [13–15]. Thus, an examination for α^0^-thal is essential for carrier couples who are at risk of conceiving a fetus with Bart’s hydrops fetalis.

A single-tube multiplex Real-time PCR with SYBR Green 1 followed by high resolution melting (HRM) analysis is currently used for the diagnosis of α^0^-thal--^SEA^ and--^THAI^ type deletions [16]. HRM analysis is simple, high-throughput, and faster than the conventional PCR since it does not require the post-PCR processing steps [17–19]. This approach would be more cost-effective if it could be processed as a single-tube multiplex PCR for the simultaneous diagnosis of several types of thalassemia. Although SYBR Green is the most widely used qPCR dye, it still has several disadvantages. One problem is that the low dye concentration required to minimize SYBR Green’s interference in the PCR can cause dye redistribution problems during DNA melting curve analysis, making it unsuitable for repeat melting, multiplexing PCR and high-resolution genotyping [20]. EvaGreen is a newly developed DNA-binding dye that has recently been used in qPCR, post-PCR, DNA melting curve analysis and several other applications [21]. Therefore, the aim of this study was to develop a system of single-tube multiplex real-time PCR using EvaGreen and HRM analysis for rapid diagnosis of α-thal--^SEA^,--^THAI^ and--^CR^ type deletions, the most frequent thalassemia large gene deletions found in Thai and Southeast Asian populations.

## Materials and methods

### DNA samples used for development of a single-tube multiplex real-time PCR with EvaGreen and HRM analysis

DNA was extracted from ethylenediaminetetraacetic acid (EDTA)-anticoagulated blood samples from 10 normal individuals and 5 heterozygotes for each α^0^-thal--^SEA^,--^THAI^ and--^CR^ type deletion using the NucleoSpin^®^ kit (Macherey-Nagel; KG., Duren, Germany), according to the manufacturer’s instructions. The extracted DNA was kept at -20°C until analysis. These DNA samples were used to set up a single-tube multiplex real-time PCR and also used as negative and positive controls, which were conducted in parallel with the tested DNA samples.

### Samples for routine molecular diagnosis of α^0^-thalassemia

From November 2022 to March 2023, whole blood and cord-blood samples collected in EDTA-containing vacutainers and amniotic fluid collected in 15 mL sterile polypropylene centrifuge tubes were sent from private and government hospitals from all regions of Thailand to the Thalassemia Laboratory, Associated Medical Sciences Clinical Service Center (AMS-CSC), Chiang Mai University, Chiang Mai, Thailand for diagnosis of α^0^-thalassemia. DNA extraction was performed using the Chelex method (Chelex^®^ 100 sodium form, Sigma, Missouri, USA) [22] and the extracted DNA was kept at -20°C until assay. In total of 2,135 samples, 2 samples were amniotic fluid and the rest was EDTA blood from subjects aged range from 1 day to 93 years old (<2 years old n = 122 (5.7%), 2–21 years old n = 279 (13.1%), and >21 years old n = 1,732 (81.2%)).

### A single-tube multiplex real-time PCR with EvaGreen and HRM analysis for diagnosis of α^0^-thal−^SEA^,--^THAI^ and−^CR^ type deletions

DNA amplification was carried out in a 20 μL reaction mixture containing 10 μL 2× Precision Melt Supermix (Bio-Rad, California, USA); primer sets specific for α^0^-thal−^SEA^,--^THAI^ and−^CR^ type deletions and the wild-type α-globin gene, which was used as the internal control; and 5 μL (1–50 ng/reaction) DNA sample. The schematic representation of these 3 deletions on the α-globin gene cluster and the position of the primers used in this study are shown in Fig 1. Nucleotide sequences and final concentrations of the primers are shown in Table 1. PCR with EvaGreen was performed using the CFX96 Touch™ Real-Time PCR Detection System (Bio-Rad, California, USA). An initial denaturation was performed at 95°C for 2 min, followed by 40 cycles at 95°C for 10 sec, 59°C for 30 sec and 72°C for 30 sec. The amplification cycles were followed by a heteroduplex formation at 95°C for 30 sec and at 60°C for 1 min. A high-resolution melting cycle was then processed from 80°C to 93°C at a rate of 0.2°C/10 sec. When the melting temperature (T_m_) is reached, double-stranded DNA is denatured and EvaGreen is released, which causes a dramatic decrease in fluorescence intensity. The rate of this change was determined by plotting the derivative of the fluorescence relative to that of the temperature (-d(RFU)/dT) using the data analysis software of the real-time PCR instrument. The temperature at which a peak occurs on the plot corresponds to the T_m_ of the DNA duplex. The result was confirmed by the conventional Gap-PCR method using positive and negative controls and samples with a positive result for each deletion [S1 Fig].

**Table 1:** Primer sequences used for single-tube multiplex real-time PCR with EvaGreen and high-resolution melting analysis.

Primers	Sequence (5′→ 3′)	Product length (bp)	Concentration (nM)
WT and SEA-F	AGA AGC TGA GTG ATG GGT CCG		250
WT-R	ACA AAC GCC CGT CCG ACT CAA	196	250
SEA-R	TGG ACT TAA GTG ATC CTC CTG CCC	134	250
Thai-F	ATT CAC ATA CCA TAC GGT TCA C		250
Thai-R	AAT TCC CCT GGA CTT GAG TG	180	250
CR-F	TAG CCT GGA GGA CAG AGT AAG		250
CR-R	CGG GTC ATG TTG AGT AGG AAT AA	335	250

Abbreviation: SEA, Southeast Asian; CR, Chiang Rai; F, Forward primer; R, Reverse primer; WT, Wild-type

## Results

### Melting curve analysis for α^0^-thal--^SEA^,--^THAI^ and--^CR^ type deletions

**Fig 2:**
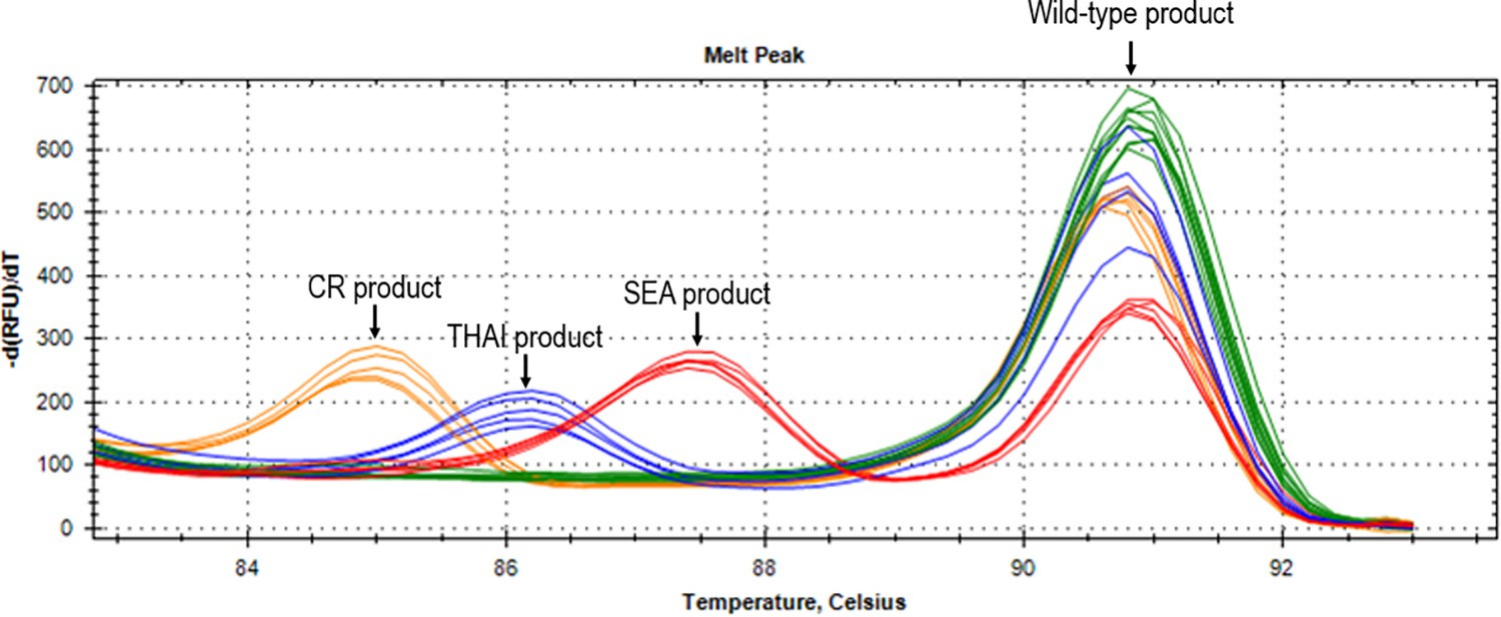
Representative results of dissociation curve analysis for α^0^-thal--^SEA^,--^THAI^ and--^CR^ type deletions.

The melt peaks (T_m_ peaks) at 85.40 ± 0.31°C, 86.50 ± 0.24°C, 87.65 ± 0.09°C, and 91.04 ± 0.20°C were specifically found in an individual who carried the α^0^-thal--^CR^,--^THAI^ and--^SEA^ type deletions and wild-type α-globin gene, respectively. The double T_m_ peaks at 85°C and 91°C were found in a sample with heterozygous--^CR^ deletion while the double T_m_ peaks at 86°C and 91°C were found in a sample with heterozygous--^THAI^ deletion and the double T_m_ peaks at 87°C and 91°C were found in a sample with heterozygous--^SEA^ deletion. The single T_m_ peak at 91°C was found in a normal individual who was negative for α^0^-thal--^CR^,--^THAI^ and--^SEA^ type deletions [Fig 2].

DNA samples were obtained from 10 normal individuals (Green) and 5 heterozygous individuals for the α^0^-thal--^SEA^ (Red), α^0^-thal--^THAI^ (Blue), and α^0^-thal--^CR^ type deletions (Orange). Abbreviations: dF/dT, derivative of the fluorescence/derivative of the temperature; SEA, Southeast Asian; CR, Chiang Rai.

### Routine diagnosis of α^0^-thal--^SEA^,--^THAI^ and--^CR^ type deletions by a single-tube multiplex real-time PCR with EvaGreen and HRM analysis

From November 2022 to March 2023, multiplex qPCR with HRM analysis was performed in 2,135 samples. Among these, α^0^-thal--^SEA^ was found in 382 (17.89%) samples, including 297 (13.91%) α^0^-thal--^SEA^ heterozygotes, 67 (3.14%) deletional HbH diseases, 16 (0.75%) nondeletional HbH diseases, and 2 (0.09%) cases of Hb Bart’s hydrops fetalis. Moreover, α^0^-thal--^THAI^ was found in 4 (0.19%) individuals comprising 3 (0.14%)--^THAI^ heterozygotes and 1 (0.05%) deletional HbH disease whereas heterozygous α^0^-thal--^CR^ was found in 4 (0.19%) individuals. The α-thal genotypes and hematological parameters for each group are shown in Table 2.

**Table 2:** Genotype of α^0^-thalassemia and hematological parameters of each group. The results of hematological parameters are shown by mean ± standard deviation.

α^0^-thalassemia genotypes	Number (%)	Hematological parameters				
		RBC counts (x 10^12^ cells/L)	Total Hb (g/dL)	PCV (L/L)	MCV (fL)	MCH (pg)
Negative for α^0^-thal (--^SEA^,--^THAI^ and--^CR^)	1,745 (81.73)	4.75 ± 1.00	11.2 ± 2.7	0.34 ± 0.07	74.2 ± 10.4	23.8 ± 4.2
α^0^-thal--^SEA^						
• α^0^-thal--^SEA^ trait (--^SEA^/αα)	297 (13.91)	5.35 ± 1.14	10.8 ± 2.4	0.35 ± 0.08	65.7 ± 6.7	20.6 ± 2.5
• Deletional HbH disease (--^SEA^/-α^3.7 or 4.2^)	67 (3.14)	4.96 ± 1.16	8.5 ± 1.7	0.29 ± 0.06	59.0 ± 6.6	17.3 ± 1.7
• Nondeletional HbH disease (--^SEA^/α^CS^α)	16 (0.75)	4.56 ± 1.36	8.5 ± 2.5	0.29 ± 0.08	65.7 ± 8.0	18.7 ± 1.9
• Hb Bart’s hydrops fetalis (--^SEA^/--^SEA^)	2 (0.09)	NA	NA	NA	NA	NA
α^0^-thal--^THAI^						
α^0^-thal--^THAI^ trait (--^THAI^/αα)	3 (0.14)	4.58 ± 0.48	9.5 ± 1.3	0.31 ± 0.04	67.5 ± 2.1	30.7 ± 0.1
Deletional HbH disease (--^THAI^/-α^3.7 or 4.2^)	1 (0.05)	5.91	9.4	0.33	55.0	15.9
α^0^-thal--^CR^ trait (--^CR^/αα)	4 (0.19)	5.42 ± 0.85	10.9 ± 1.1	0.35 ± 0.04	65.4 ± 3.7	20.2 ± 1.5

Normal ranges for the measurements presented in the table are as follows: RBC counts 4.0–5.4 x 10^12^/L; Total Hb 12.0–18.0 g/dL; PCV 0.35–0.55 L/L; MCV 80–94 fL; MCH 26–32 pg

SEA, Southeast Asian; CR, Chiang Rai; CS, Constant Spring; 3.7 or 4.2, 3.7 or 4.2 kb deletions; NA, Not available; RBC, Red blood cell; Hb, Hemoglobin; PCV, Packed cell volume; MCV, Mean corpuscular volume; MCH, Mean corpuscular hemoglobin

## Discussion

Real-time PCR with SYBR Green 1 and HRM analysis are currently used for the identification of thalassemia mutations [16–19, 23]. This technique does not require fluorescently labeled probes or separation steps. Therefore, it offers additional advantages, including effortful, a reduced turnaround time, and a decreased risk of carryover contamination. However, only a relatively low SYBR Green concentration (e.g. <0.5 μM) can be used in the reaction because of its high tendency to inhibit PCR and promote mispriming [24]. It has been reported that SYBR Green failed to detect multiplex PCR products by displaying only a single melt peak even though gel electrophoresis revealed multiple products [24]. In addition, an insufficient concentration of dye led to SYBR Green migration from the low-melting to the high-melting amplicons during the DNA melting process. Therefore, a low SYBR Green concentration may not only compromise PCR signal strength but may also result in DNA melting curve data being unreliable due to the dye redistribution during melting curve analysis [20]. The use of the SYBR Green master mix is not cost effective, especially for single gene analysis. To reduce the cost and complexity of diagnosing multiple type deletions of α^0^-thal (--^SEA^,--^THAI^ and--^CR^), we developed a system of single-tube multiplex real-time PCR with EvaGreen and HRM analysis. The EvaGreen is a newly developed DNA-binding dye, and has recently been used in qPCR and related applications. The dye can be used at a relatively high concentration (i.e., 1.34 μM), thus permitting a more robust PCR signal as well as a stronger and sharper DNA melt peak. A relatively high dye concentration in qPCR also eliminates the dye redistribution problem, making the dye suitable for HRM application in the closed-tube format. A specific peak height was observed for each type of α^0^-thal assessed using this approach. This technique can be used for diagnosing heterozygotes, homozygotes, and compound heterozygotes of α^0^-thal--^SEA^,--^THAI^ and--^CR^ type deletions. In addition, the results interpreted using single-tube multiplex real-time PCR with EvaGreen and HRM analysis were completely consistent with those derived using conventional gap-PCR [S1 Fig]. Moreover, based on a system of single-tube multiplex real-time PCR with EvaGreen and HRM analysis, we found that the detection rates of α^0^-thal--^SEA^,--^THAI^ and--^CR^ type deletions were comparable to those reported previously [25]. The first case of Hb Bart’s hydrops fetalis caused by α^0^-thal−^SEA^/--^CR^ was reported in 2020 [10]. To date, 21 cases of α^0^-thal--^CR^ type deletion have been diagnosed throughout Thailand. Most of them (19 cases) were found in northern region; 13 in Chiang Mai, 5 in Phayao and 1 in Mae Hong Son. Other 2 cases were found in central and southern regions (Nakhon Pathom and Songkhla provinces, respectively) [10, 11, 25]. Although the highest frequency of α^0^-thal--^CR^ was found in northern Thailand, it can be found across the country. Therefore, these results could be used to guide laboratory setup for a prevention and control program for severe α^0^-thal in this region.

In conclusion, our results indicate that single-tube multiplex real-time PCR with EvaGreen and HRM analysis for diagnosis of α^0^-thal--^SEA^,--^THAI^ and--^CR^ type deletions is as effective as a conventional method. This method would significantly reduce the cost and complexity of screening for these α^0^-thal type deletions. Thus, single-tube multiplex real-time PCR with EvaGreen and HRM analysis may be used as an alternative for routine clinical diagnosis of α^0^-thal and may be especially useful for genetic counseling in prevention and control programs for severe thalassemias, including HbH disease and Hb Bart’s hydrops fetalis, in regions with a high prevalence of α-thalassemia.

## Supporting information

S1 Fig Result of conventional Gap-PCR method for α^0^-thal--^SEA^,--^THAI^ and--^CR^ heterozygote DNA samples. M: 100 bp DNA ladder, Lane 1: Normal DNA sample, Lane 2: α^0^-thal--^SEA^ heterozygote, Lane 3: α^0^-thal--^THAI^ heterozygote, Lane 4: α^0^-thal--^CR^ heterozygote.
(TIF)

S1 Data (XLSX)
