# Supplementary figures and images for: Single-tube multiplex real-time PCR with EvaGreen and high-resolution melting analysis for diagnosis of α0-thalassemia--SEA,--THAI, and--CR type deletions
Source: PLoS One. 2023 Nov 6;18(11):e0293838. doi: 10.1371/journal.pone.0293838 (PMC10627449; doi:10.1371/journal.pone.0293838)

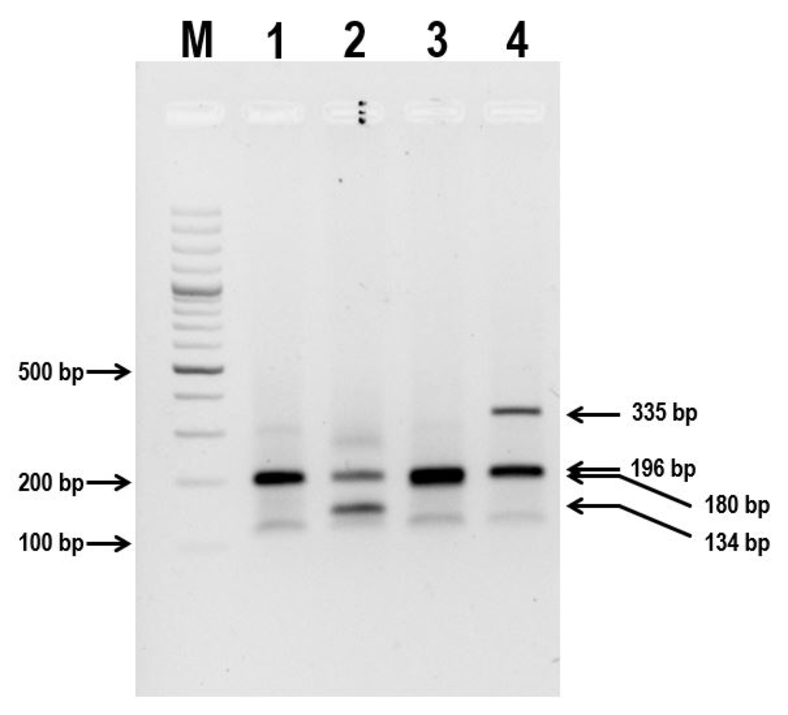

Supplement: S1 Fig — M: 100 bp DNA ladder, Lane 1: Normal DNA sample, Lane 2: α0-thal--SEA heterozygote, Lane 3: α0-thal--THAI heterozygote, Lane 4: α0-thal--CR heterozygote. (TIF) [file pone.0293838.s001.tif]
